# Temporal changes in tolerance of uncertainty among medical students: insights from an exploratory study

**DOI:** 10.3402/meo.v20.28285

**Published:** 2015-09-08

**Authors:** Paul K. J. Han, Daniel Schupack, Susannah Daggett, Christina T. Holt, Tania D. Strout

**Affiliations:** 1 Center for Outcomes Research and Evaluation, Maine Medical Center, Portland, ME, USA; 2 Internal Medicine Residency Program, Mayo School of Graduate Medical Education, Mayo Clinic, Rochester, MN; 3 Department of Family Medicine, Maine Medical Center, Portland, ME, USA; 4 Department of Emergency Medicine, Maine Medical Center, Portland, ME, USA

**Keywords:** ambiguity, medical students, tolerance, uncertainty

## Abstract

**Background:**

Physicians’ tolerance of uncertainty (TU) is a trait potentially associated with desirable outcomes, and emerging evidence suggests it may change over time. Past studies of TU, however, have been cross-sectional and have not measured tolerance of the different, specific types of uncertainty that physicians confront. We addressed these limitations in a longitudinal exploratory study of medical students.

**Methods:**

At the end of medical school (Doctor of Medicine degree) Years 1 and 4, a cohort of 26 students at a US medical school completed measures assessing tolerance of different types of uncertainty: 1) complexity (uncertainty arising from features of information that make it difficult to comprehend); 2) risk (uncertainty arising from the indeterminacy of future outcomes); and 3) ambiguity (uncertainty arising from limitations in the reliability, credibility, or adequacy of information). Change in uncertainty-specific TU was assessed using paired *t*-tests.

**Results:**

Between Years 1 and 4, there was a significant decrease in tolerance of ambiguity (*t*=3.22, *p*=0.004), but no change in students’ tolerance of complexity or risk.

**Conclusions:**

Tolerance of ambiguity – but not other types of uncertainty – decreases during medical school, suggesting that TU is a multidimensional, partially mutable state. Future studies should measure tolerance of different uncertainties and examine how TU might be improved.

Physicians’ tolerance of uncertainty (TU) has become a matter of growing interest among medical educators. Emerging research suggests that TU varies among medical trainees and physicians and that greater TU is associated with various outcomes including greater comfort dealing with grief, loss, and death; reduced fear of medical error and malpractice, and reduced propensity to order diagnostic tests (1, 2). The desirability of such outcomes raises the question of whether TU is a fixed – as opposed to a mutable – characteristic that ‘can be taught and nurtured’ (1). Past studies of TU among medical trainees, however, have yielded conflicting answers to this question. Some have shown a positive relationship between TU and higher levels of medical education (3–5), suggesting that TU changes over time, while other studies have shown no relationship (6, 7).

These studies, however, have been limited by their cross-sectional design as well as important conceptual and measurement problems. Most studies have not distinguished between the specific types of uncertainty that physicians confront – and that constitute the putative objects of their tolerance – but instead have treated uncertainty as a monolithic phenomenon (1). Past studies have correspondingly employed various measures of TU that focus on physicians’ *responses to* uncertainty in general, for example, emotional ‘stress from uncertainty’, ‘reluctance to disclose uncertainty to patients’ (8, 9), and ‘preference for highly structured training environs’ (5).

Yet uncertainty represents a multidimensional phenomenon consisting of the conscious awareness of ignorance arising from distinct sources: probability, ambiguity, and complexity (Fig. 1) (10). Probability, or ‘risk’, refers to the indeterminacy of future outcomes; ambiguity – a construct from the decision theory literature – refers to limitations in the reliability, credibility, or adequacy of information (11); whereas complexity refers to features of information that make it difficult to comprehend. Behavioral research has shown that uncertainties arising from these different sources have unique effects and are associated with varying levels of tolerance among individuals (10, 12–15).

**Fig. 1:**
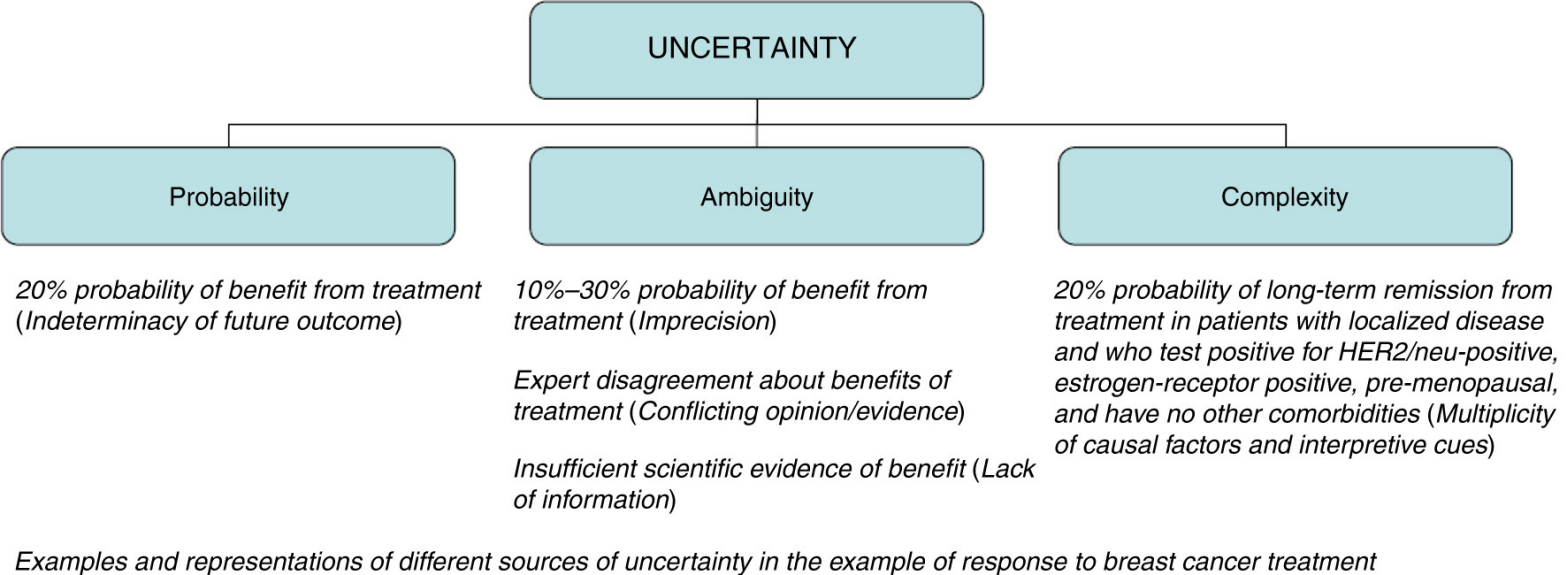
Sources of uncertainty in health care. Adapted from Han et al. (10).

However, most past studies of TU have not distinguished these uncertainties, and indeed have used the terms ‘uncertainty’ and ‘ambiguity’ interchangeably (1). Only two studies have attempted to explicitly measure tolerance of different types of uncertainty among medical practitioners or trainees (4, 6, 8). One study measured practicing primary care physicians’ tolerance of both general uncertainty (using the Tolerance for Ambiguity (TFA) (6) and Physician Reaction to Uncertainty (PRU) (8) scales, both of which arguably measure tolerance of complexity) and of risk (using the Pearson Risk Attitude (PRA) scale (16)) and found that both tolerance of general uncertainty (measured only by the PRU scale) and risk predicted physician resource use (17). Another study assessed both primary care physicians’ tolerance of general uncertainty (using the PRU) and risk (using the PRA) and found that each predicted use of different literature-searching strategies (18). These studies, however, did not measure tolerance of ambiguity (as defined in decision theory terms) and no study has assessed tolerance of different uncertainties among trainees.

This lack of conceptual and measurement specificity may account for the historically inconsistent findings regarding the development of TU among medical trainees, and the overarching objective of the current research was to explore this possibility. We undertook an exploratory study designed to longitudinally measure medical students’ specific tolerance of risk, ambiguity, and complexity. Our goal was to obtain proof-of-principle evidence on whether students’ tolerance of different types of uncertainty change over time, justifying a multidimensional approach to measuring – and potentially intervening upon – this attribute.

## Methods

### Study population and design

The study population consisted of the ‘Maine Track’ (MT) cohort (Class of 2014) of Tufts University School of Medicine: approximately 36/200 students in each class who are interested in rural health care and receive most clinical training at Maine rather than Boston hospitals. Survey measures, described below, were administered to MT students at the end of both Year 1 (2011) and Year 4 (2014) of medical school.

### Measures

Three uncertainty-specific TU measures were completed by the cohort (Table 1). *Tolerance for Ambiguity* (TFA) is a seven-item measure designed to assess tolerance of general uncertainty in life, although its items focus primarily on uncertainty arising from complexity (6). The scale has acceptable reliability (*α*=0.75), and higher tolerance of ambiguity has been shown to predict medical students’ specialty choice and physician interest in ordering a genetic test of unknown clinical utility (6).

**Table 1:** Uncertainty tolerance measures used in the current study^a^

A. Tolerance for Ambiguity (TFA) (6)
1. It really disturbs me when I am unable to follow another person's train of thought.
2. If I am uncertain about the responsibilities involved in a particular task, I get very anxious.
3. Before any important task, I must know how long it will take.
4. I don't like to work on a problem unless there is a possibility of getting a clear-cut and unambiguous answer.
5. The best part of working on a jigsaw puzzle is putting in that last piece.
6. I am often uncomfortable with people unless I feel that I can understand their behavior.
7. A good task is one in which what is to be done and how it is to be done are always clear.
B. Pearson Risk Attitude (PRA) (16)
1. I enjoy taking risks.
2. I try to avoid situations that have uncertain outcomes.^b^
3. Taking risks does not bother me if the gains involved are high.
4. I consider security an important element in every aspect of my life.^b^
5. People have told me that I seem to enjoy taking chances.
6. I rarely, if ever, take risks when there is another alternative.^b^
C. Ambiguity Aversion in Medicine (AA-Med) (19)
1. I would not have confidence in a medical test or treatment if experts had conflicting opinions about it.
2. Conflicting expert opinions about a medical test or treatment would make me upset.
3. I would not be afraid of trying a medical test or treatment even if experts had conflicting opinions about it.^b^
4. If experts had conflicting opinions about a medical test or treatment, I would still be willing to try it.^b^
5. I would avoid making a decision about a medical test or treatment if experts had conflicting opinions about it.

a All items rated on six-point Likert scale ranging from ‘Strongly Disagree’ to ‘Strongly Agree’.

b Reverse-scored item.

The *Pearson Risk Attitude* (PRA) scale is a six-item measure of TU arising from probability or risk. The PRA scale has acceptable reliability (*α*=0.71), and lower risk tolerance among emergency room physicians has been shown to be associated with higher rates of admission for patients with acute chest pain (16).

The *Ambiguity Aversion in Medicine* (AA-Med) scale is a six-item measure of aversion to ambiguity (19), conceptualized specifically in decision theory terms, as uncertainty regarding the reliability, credibility, or adequacy of risk information. The scale has acceptable reliability (*α*=0.73), and higher ambiguity aversion has been shown to predict lower interest among laypersons in a hypothetical ambiguous cancer screening test, and pessimistic appraisals of the benefits and harms of cancer screening interventions (19–21). Originally designed for laypersons, the scale was adapted for the current study by omitting a single item, ‘Conflicting expert opinions about a medical test or treatment would lower my trust in the experts’.

### Data analyses

Descriptive analyses were conducted and Cronbach's alpha was calculated to evaluate measure reliability at both time points. Associations between TU measures were evaluated using Pearson's correlations, and change in TU assessed by each measure was evaluated using paired (matched) *t*-tests. As a nonparametric sensitivity analysis given the study's small sample size, change in TU was also evaluated using the Wilcoxon signed ranks test.

## Results

Thirty-two students completed the Year 1 measures, and 26 (79%) completed the Year 4 measures. The cohort was 50% male, 92% Caucasian, with a mean age of 24 years at Year 1, mean Medical College Admissions Test score of 32.03 and mean college grade point average of 3.54.

Scores on the PRA, TFA, and AA-Med scales were normally distributed. There was a significant correlation between Year 1 and Year 4 scores for the PRA, but not for the TFA and AA-Med scales (Table 2). There were moderate-sized correlations between Year 4 PRA and TFA scale scores and between Year 1 TFA and AA-Med scale scores, but no other significant correlations, suggesting the scales measure distinct constructs. Scale reliability was acceptable (*α*≥0.70) for all scales but were relatively low for the AA-Med scale administered during Year 4 (*α*=0.59) (Table 3).

**Table 2:** Pearson correlations between different uncertainty tolerance measures

	PRA1	PRA4	TFA1	TFA4	AAM1	AAM4
Pearson Risk Attitude – Year 1 (PRA1)	–	0.664**	−0.103	−0.172	−0.109	0.021
Pearson Risk Attitude – Year 4 (PRA4)		–	−0.068	−0.445*	−0.178	−0.167
Tolerance for Ambiguity – Year 1 (TFA1)			–	0.137	0.427*	−0.115
Tolerance for Ambiguity – Year 4 (TFA4)				–	−0.102	0.162
Ambiguity Aversion in Medicine – Year 1 (AAM1)					–	0.385
Ambiguity Aversion in Medicine – Year 4 (AAM4)						–

* *p*<0.05,

** *p*<0.01 for two-tailed test of significance.

**Table 3:** Change in uncertainty tolerance scores between medical school years 1 and 4

	Year 1^a^	*α*	Year 4^a^	*α*	*t*	*p*
Tolerance for Ambiguity (TFA)	3.81 (0.78)	0.77	3.63 (0.72)	0.74	0.92	0.37
Pearson Risk Attitude (PRA)	2.82 (0.80)	0.85	2.71 (0.81)	0.75	0.86	0.40
Ambiguity Aversion in Medicine (AA-Med)	3.05 (0.60)	0.70	2.65 (0.56)	0.59	3.22	**0.004**

a All scale scores reported as mean (standard deviation); score range 1–6; higher scores for TFA and AA-Med indicate lower uncertainty tolerance; higher scores for PRA indicate higher uncertainty tolerance.

Bolded value indicates *p*<0.05.

No temporal differences in either tolerance of complexity (TFA scores) or tolerance of risk (PRA scores) were noted (Table 3). However, there were significant differences in ambiguity aversion (AA-Med scores). AA-Med scores were significantly lower at Year 4 than Year 1, indicating a temporal decrease in ambiguity aversion. Nonparametric analyses yielded the same pattern of results, demonstrating a significant temporal difference in AA-Med scores only (*Z*=2.80, *p*=0.005).

## Discussion

The current study – to our knowledge, the first to explore TU among medical students both longitudinally and using multiple uncertainty-specific measures – demonstrated a significant temporal decrease in students’ aversion to ambiguity but no change in their tolerance of either risk or complexity. These findings are clearly preliminary given the study's small sample size; however, they have important implications.

The overarching implication is that TU is not a monolithic, static trait but a multifaceted, dynamic state (10), and that aversion to – or conversely, tolerance of – ambiguity is a distinguishable TU subtype that is more malleable than tolerance of either complexity or risk. More research is needed to confirm these findings, since our study may have had insufficient power to detect change in all TU subtypes. However, our findings are consistent with past cross-sectional studies (6, 7), as well as emerging evidence on the evolution of medical trainees’ beliefs about the nature of knowledge. Medical school is a time in which students are exposed, often for the first time, to the ambiguity pertaining to medical tests and treatments, and this exposure may promote more sophisticated epistemological beliefs and greater tolerance of this ambiguity (4, 22). In particular, specific aspects of the medical school curriculum – for example, training in evidence-based medicine and critical appraisal of the medical literature – may heighten students’ awareness of the insufficient and conflicting scientific evidence underlying much of medical practice, and the need to accept these limitations. In contrast, the medical school curriculum *per se* does not aim to promote tolerance of risk or complexity. These TU subtypes, furthermore, are not specific to medicine and may be more fully developed – and resistant to change – by the time students reach medical school.

More research is needed to test these and alternative explanations of our findings and to address several study limitations. Our study was conducted at a single institution among a small cohort of students whose interest in rural health care may have influenced their baseline level and subsequent development of TU. The study assessed TU at only two time points using only three specific TU measures. Additional longitudinal studies – using larger, multiinstitution samples and multiple measures of TU subtypes – are needed to confirm our findings. Further research is also needed to examine how other unmeasured characteristics, including students’ medical knowledge and skills, might influence TU.

Despite these limitations, our study provides seminal proof-of-principle evidence that tolerance of ambiguity – an important subtype of TU unmeasured in previous studies – changes during medical education. This finding has practical implications for researchers and educators. It raises the need to understand and measure TU in an uncertainty-specific manner. It suggests that tolerance of ambiguity indeed ‘can be taught and nurtured’ (1) and calls for further work to develop deliberative approaches to this task and to assess the outcomes of greater ambiguity tolerance for physicians and patients (1, 23). Our study endorses the value of such efforts.
